# Biofabrication of prevascularized spheroids for bone tissue engineering by fusion of microvascular fragments with osteoblasts

**DOI:** 10.3389/fbioe.2024.1436519

**Published:** 2024-09-10

**Authors:** Selina Wrublewsky, Jessica Schultz, Tekoshin Ammo, Caroline Bickelmann, Wolfgang Metzger, Thomas Später, Tim Pohlemann, Michael D. Menger, Matthias W. Laschke

**Affiliations:** ^1^ Institute for Clinical and Experimental Surgery, Saarland University, Homburg, Germany; ^2^ Department of Trauma, Hand, and Reconstructive Surgery, Saarland University, Homburg, Germany; ^3^ Department of Molecular, Cell and Systems Biology, University of California, Riverside, Riverside, CA, United States

**Keywords:** microvascular fragments, osteoblasts, spheroid, bone, tissue engineering, vascularization

## Abstract

**Introduction:**

Spheroids are promising building blocks for scaffold-free bone tissue engineering. Their rapid vascularization is of major importance to guarantee their survival after transplantation. To achieve this, we herein introduce the biofabrication of prevascularized spheroids by fusion of adipose tissue-derived microvascular fragments (MVF) with osteoblasts (OB).

**Methods:**

For this purpose, 200 MVF from donor mice and 5,000, 10,000 or 20,000 murine OB (MC3T3-E1) were co-cultured in a liquid overlay system for 3 days to generate OB + MVF spheroids. OB mono-culture spheroids served as controls.

**Results and discussion:**

During the generation process, the diameters of all spheroids progressively decreased, resulting in compact, viable spheroids of homogeneous sizes. MVF promoted the maturation of spheroids containing 5,000 OB, as shown by an accelerated decline of cell proliferation due to contact inhibition. Moreover, MVF most effectively reassembled into new microvascular networks within these small spheroids when compared to the other spheroid types, indicating the most beneficial MVF to OB ratio. Accordingly, these spheroids also showed a high angiogenic sprouting activity *in vitro*. In contrast to OB spheroids, they further rapidly vascularized *in vivo* after transplantation into dorsal skinfold chambers. This was caused by the interconnection of incorporated MVF with surrounding blood vessels. These findings indicate that OB + MVF spheroids may be suitable for bone tissue engineering, which should be next tested in appropriate *in vivo* bone defect models.

## 1 Introduction

Critical-sized bone defects can be caused by injury, infection or tumor resection. The treatment of such large defects represents a major challenge in trauma and orthopedic surgery. For this purpose, bone autografts, allografts or synthetic bone substitutes are currently used in clinical practice (Wang and Yeung, 2017). However, their implantation is frequently associated with several disadvantages. These include limited graft availability, donor site morbidity and infection (Wang and Yeung, 2017; Baldwin et al., 2019). To overcome these problems, strong efforts have been put into the development of scaffold-based and scaffold-free bone tissue engineering strategies during the last years (Maia et al., 2022).

Spheroids are promising building blocks for scaffold-free tissue engineering (Prabhakaran et al., 2023). Due to their three-dimensional (3D) structure and close cell-cell contacts, they exhibit tissue-like biological properties. Therefore, their cell viability, proliferative activity and physiological metabolic function are markedly improved when compared to two-dimensional single cell systems (Laschke and Menger, 2017). Accordingly, spheroids consisting of osteoblasts (OB) or mesenchymal stem cells (MSC) have already been shown to biomineralize *in vitro* and effectively promote bone formation *in vivo* (Fennema et al., 2018; Itel et al., 2018; Ahmad et al., 2020).

A major prerequisite for the long-term viability and bone-forming capacity of grafted spheroids is the rapid establishment of a sufficient blood supply, because they cannot only survive by diffusion of oxygen and nutrients. To address this issue, prevascularized spheroids by co-culturing OB or MSC with endothelial cells have been generated (Rouwkema et al., 2006; Walser et al., 2013; Heo et al., 2019). After transplantation, these spheroids exhibited an accelerated and improved vascularization when compared to non-prevascularized controls (Walser et al., 2013). However, the use of primary endothelial cells or pluripotent stem cell-derived endothelial cells is difficult to broadly implement into clinical practice (Hewett, 2016; Ikuno et al., 2017). Moreover, these cells do not necessarily develop into complete functional microvascular networks when incorporated into OB spheroids. Therefore, a more promising approach may be the fusion of microvascular fragments (MVF) with OB. This approach has previously been described for the generation of prevascularized pseudoislets in diabetes therapy (Nalbach et al., 2021b).

In contrast to single endothelial cells, MVF bear the major advantage that they are already functional arteriolar, capillary and venous vessel segments (Laschke and Menger, 2015). They can be easily isolated in large quantities by enzymatic digestion of adipose tissue (Frueh et al., 2017). Accordingly, MVF rapidly connect with each other and with blood vessels of the surrounding host tissue after transplantation (Laschke et al., 2021). Hence, MVF have been proven in various experimental settings to be highly potent prevascularization units for implanted scaffolds and tissue constructs (Laschke and Menger, 2022).

For these reasons, we herein introduce the biofabrication of prevascularized spheroids for bone tissue engineering by fusion of MVF with OB in a liquid overlay culture. The morphology, viability, proliferation and angiogenic activity of these co-culture spheroids were analyzed *in vitro* in comparison to OB mono-culture spheroids. Moreover, we assessed the *in vivo* vascularization of OB and OB+ MVF spheroids in a mouse dorsal skinfold chamber model by means of intravital fluorescence microscopy and immunohistochemistry.

## 2 Materials and methods

### 2.1 Material

Trypsin, α-Minimum Essential Medium (α-MEM) without ascorbic acid, Tissue-Tek and Dulbeccos Modified Eagle Medium (DMEM) were purchased from Thermo Fisher Scientific (Karlsruhe, Germany). Hexamethyldisilazane (HMDS) was purchased from Carl Roth GmbH + Co KG (Karlsruhe, Germany). IVISbrite D-Luciferin Potassium Salt Bioluminescent Substrate was purchased from Revvity (Hamburg, Germany). Fluorescein isothiocyanate (FITC)-labeled dextran 150,000, collagenase IAS, Hoechst 33342, dexamethasone, nuclear fast red solution and penicillin-streptomycin were purchased from Sigma-Aldrich (Taufkirchen, Germany). HepatoQuick^®^ was purchased from Roche (Basel, Switzerland). Fetal calf serum (FCS) was purchased from Biochrom GmbH (Berlin, Germany). Collagen was purchased from Advanced BioMatrix (Carlsbad, United States). Hematoxylin was purchased from Morphisto (Offenbach am Main, Germany). β-Glycerophosphate was purchased from Calbiochem (Darmstadt, Germany). Ascorbic acid was purchased from Fluka (Buchs, Switzerland).

### 2.2 Antibodies

The anti-CD31 antibody (1:50; DIA310) was purchased from Dianova (Hamburg, Germany). The anti-green fluorescent protein (GFP; 1:50; 600-101-215) antibody was purchased from Rockland Immunochemicals (Pottstown, United States). The anti-cleaved caspase-3 (casp-3; 1:300; 9664S) and anti-Ki67 (1:300; 9129) antibodies were purchased from Cell Signaling Technology (Leiden, Netherlands). The anti-rat IgG Alexa Fluor 555 (1:100; A21434) and anti-goat IgG Alexa Fluor 488 (1:100; A11055) antibodies were purchased from Thermo Fisher Scientific. The peroxidase-labeled anti-rabbit antibody (1:100; NIF 824) was purchased from GE Healthcare (Freiburg, Germany).

### 2.3 Cell culture

Murine OB (MC3T3-E1) were purchased from American Type Culture Collection^®^ (ATCC; Manassas, United States, Cat# CRL-2593, Lot# 70009744) and were cultivated in α-MEM (10% (v/v) FCS) at 37°C under a humidified 95%–5% (v/v) mixture of air and CO_2_.

### 2.4 Animals

C57BL/6J wildtype (WT), FVB-Tg(CAG-luc-GFP)L2G85Chco/J and C57BL/6-Tg(CAG-EGFP)1Osb/J (Charles River, Köln, Germany) mice with a body weight of 25–35 g served as fat donors for the isolation of MVF. C57BL/6J WT mice with a body weight of 22–27 g were used for the preparation of the dorsal skinfold chamber. The animals were maintained on a standard 12/12 h day/night cycle. Water and standard pellet chow (Altromin, Lage, Germany) were provided *ad libitum*.

All experiments were performed according to the German legislation on protection of animals and the National Institutes of Health (NIH) Guide for the Care and Use of Laboratory Animals (Institute of Laboratory Animal Resources, National Research Council, Washington DC, United States). The experiments were approved by the local governmental animal protection committee (permission number: 34/2020).

### 2.5 MVF isolation

Mice were anesthetized by an intraperitoneal (i.p.) injection of ketamine (100 mg/kg body weight) and xylazine (12 mg/kg body weight) and euthanized by cervical dislocation. MVF were isolated by mechanical and enzymatic digestion of the visceral fat pads of donor mice. The fat pads were harvested through a midline laparotomy, washed in 1 x Hanks’ Balanced Salt Solution (HBSS) and mechanically minced by means of scissors. Subsequently, the minced fat (1 mL) was digested in collagenase IAS (5 U/mL in 1 × HBSS) for 10 min under vigorous stirring at 1,000 rpm and 37°C in a ThermoMixer C (Eppendorf, Hamburg, Germany). The digestion was stopped by adding 5 mL DMEM (10% (v/v) FCS, 100 U/mL penicillin and 0.1 mg/mL streptomycin). The layer containing MVF was separated from the overlaying fat layer by gravity. After discarding the fat layer, the MVF layer was filtrated through a 500 µm filter and washed twice with 5 mL DMEM by centrifugation at 1,000 g for 5 min at room temperature. Finally, the resulting MVF pellet was resuspended in 1 mL DMEM and the isolated MVF were counted in a Neubauer counting chamber before combining them with OB.

### 2.6 Generation of OB and OB + MVF spheroids

OB were fused with or without MVF to OB or OB + MVF spheroids by means of the liquid overlay technique in a 96-well plate covered with 1% agarose (Nalbach et al., 2021b). The wells were equilibrated with culture medium for 1 h at 37°C prior to cell seeding. In a next step, 5,000, 10,000 or 20,000 OB were seeded per well and cultivated for 3 days at 37°C and 5% CO_2_. To form OB + MVF spheroids, identical amounts of OB were co-cultured with 200 MVF. After 3 days of cultivation, the spheroids were harvested and used for further experiments. Their diameters were measured by analyzing bright field images using ImageJ software (NIH, Bethesda, United States).

### 2.7 Scanning electron microscopy

The surface topography of OB and OB + MVF spheroids was characterized by scanning electron microscopy. For this, spheroids were washed twice in phosphate-buffered saline (PBS) and fixed in 2% (v/v) glutardialdehyde in 0.1 M cacodylate buffer for 10 min at room temperature. Subsequently, the spheroids were washed three times with 0.1 M cacodylate buffer and dehydrated in an ascending ethanol series (70%, 80%, 90%, 96%, 100%). The dehydration was completed by incubation in HMDS, starting with two incubations in a 1:1 mixture of ethanol (100%) and HMDS, followed by pure HMDS, and finally an overnight incubation in HMDS to allow its complete evaporation. After sputtering, the dried samples were transferred to conductive carbon adhesive tabs and analyzed in a FEI XL 30 ESEM FEG scanning electron microscopic device (FEI, Hillsboro, United States).

### 2.8 Sprouting assay and bioluminescence imaging

The angiogenic activity of OB and OB + MVF (from FVB-Tg(CAG-luc-GFP)L2G85Chco/J mice) spheroids was determined by a sprouting assay, as previously described in detail (Nalbach et al., 2021b), in combination with bioluminescence imaging. Briefly, OB and OB + MVF spheroids were collected and resuspended in a collagen solution to transfer them into prewarmed 24-well plates (Heiss et al., 2015). After 45 min, the collagen gel was covered with DMEM (10% (v/v) FCS, 100 U/mL penicillin and 0.1 mg/mL streptomycin) and the spheroids were incubated for 5 days at 37°C and 5% CO_2_ for daily analyses. The sprouting spheroids were first visualized by a BX60F microscope (Olympus, Hamburg, Germany) and their sprouting capacity was assessed by measuring the length and number of sprouts by FIJI software (NIH). Subsequently, the visualization of MVF luciferase activity was performed by means of bioluminescence imaging. For this purpose, 1.5 mg/mL IVISbrite D-Luciferin Potassium Salt Bioluminescent Substrate was added to the culture medium. After an incubation period of 5 min to ensure the distribution of the substrate, the spheroid-loaded 24-well plates were placed inside an IVIS Spectrum *In Vivo* Imaging System (Perkin Elmer, Waltham, United States) and imaged using the Living Image software (version 4.7.3; Perkin Elmer). Bioluminescence was detected by drawing a region of interest (ROI) over each well. Within the chosen ROI, the total flux was measured in photons/second (p/s).

### 2.9 Osteogenic differentiation

For the osteogenic differentiation of spheroids, the medium was removed on day 3 of spheroid generation and culture medium supplemented with 10 nM dexamethasone, 50 μg/mL ascorbic acid and 10 mM β-glycerophosphate was added. A medium change was performed every 2 days during the differentiation period of 18 days.

### 2.10 Dorsal skinfold chamber model

Mice were anesthetized by an i.p. injection of ketamine (100 mg/kg body weight) and xylazine (12 mg/kg body weight). For peri-operative pain prevention, the animals additionally received a subcutaneous injection of 10 mg/kg carprofen (Rimadyl^®^; Zoetis Deutschland GmbH, Berlin, Germany). The dorsal skinfold chamber was prepared, as described previously in detail (Laschke and Menger, 2016). Two symmetrical titanium frames were fixed on their extended dorsal skinfold, resulting in the doubling of the skin in two layers. One layer, including cutis, subcutis and the retractor muscle, was completely removed in a circular area of 15 mm in diameter. This area was then covered by a removable cover slip and affixed by a snap ring providing direct microscopic access to the microcirculation of the chamber tissue. After the procedure, the animals were allowed to recover for 48 h.

Thereafter, the mice were anesthetized again by an i.p. injection of ketamine (100 mg/kg body weight) and xylazine (12 mg/kg body weight), the cover glass was removed and the tissue was carefully washed with saline. Subsequently, 4 OB and 4 OB + MVF (from C57BL/6-Tg (CAG-EGFP)1Osb/J mice) spheroids were transplanted onto the exposed striated muscle tissue. Finally, the chamber was sealed with a new cover slip for repeated intravital fluorescent microscopic analyses.

### 2.11 Intravital fluorescence microscopy

The anesthetized dorsal skinfold chamber-equipped mice received a retrobulbary intravenous injection of 0.05 mL FITC-labeled dextran (5%) for plasma staining on day 0 (day of spheroid transplantation), 3, 6, 10 and 14. Then, the dorsal skinfold chamber was positioned under a fluorescence microscope (Zeiss, Oberkochen, Germany) and the microscopic images were recorded for off-line evaluation by means of the computer-assisted image analysis system CapImage (version 8.5; Zeintl, Heidelberg, Germany). The vascularized area (mm^2^), functional microvessel density (cm/cm^2^) and take rate (%), i.e., the number of OB or OB + MVF spheroids exhibiting perfused microvessels on day 14 in relation to the transplanted spheroids on day 0, were assessed, as previously described in detail (Ampofo et al., 2015). In addition, we measured the diameter (µm), centerline red blood cell (RBC) velocity (µm/s) and volumetric blood flow (pL/s) of 4–8 individual microvessels within the grafts (Ampofo et al., 2015).

### 2.12 Histology and immunohistochemistry

For the preparation of histological sections, the dorsal skinfold chamber-equipped mice were euthanized by cervical dislocation immediately after the last microscopy on day 14. The dorsal skinfold chamber tissue was then excised and fixed for 24 h in 4% paraformaldehyde (PFA). For additional *in vitro* analyses, freshly generated OB and OB + MVF spheroids were incubated for 45 min at 37°C in 100 µL HepatoQuick^®^, 50 µL human citrate plasma and 10 µL 10% CaCl_2_ solution. The resulting clot was also fixed for 24 h in 4% PFA. The PFA-fixed specimens were embedded in paraffin and 3-μm-thick sections were cut.

The sections were stained with the indicated primary antibodies and visualized by their corresponding secondary antibodies. Cell nuclei were stained with Hoechst 33342 for fluorescence microscopy and with hematoxylin for bright field microscopy. The sections were analyzed by means of fluorescence microscopy and bright field microscopy (BX60F; Olympus). Positively stained cells were assessed by FIJI software (NIH).

For von Kossa staining, spheroids were fixed for 1 h in 4% PFA. The fixed spheroids were embedded in Tissue-Tek, 3-μm-thick cryosections were cut and a freshly prepared silver nitrate solution (2.5% in Aqua dest.) was added for 20 min in the dark. Silver ions in mineralized areas of spheroids were reduced by adding a 0.5% hydroquinone solution in Aqua dest. Finally, the chemical reaction was stopped by fixing in a 5% sodium thiosulfate solution in Aqua dest. Cell nuclei were stained with 0.1% nuclear fast red solution for 5 min. The sections were analyzed by means of bright field microscopy (BX60F; Olympus).

### 2.13 Statistical analysis

The statistical analysis was performed by means of GraphPad Prism software 10. Differences between two groups were analyzed by an unpaired Student’s t-test after testing the data for normal distribution and equal variance. In case of non-parametric data, a Mann-Whitney rank sum test was used. To test for time effects within each experimental group, ANOVA for repeated measurements was applied. This was followed by the Turkey *post hoc* test. Values are shown as mean ± SEM. Statistical significance was accepted for *p* < 0.05.

## 3 Results

### 3.1 Generation and size of spheroids

**FIGURE 1:**
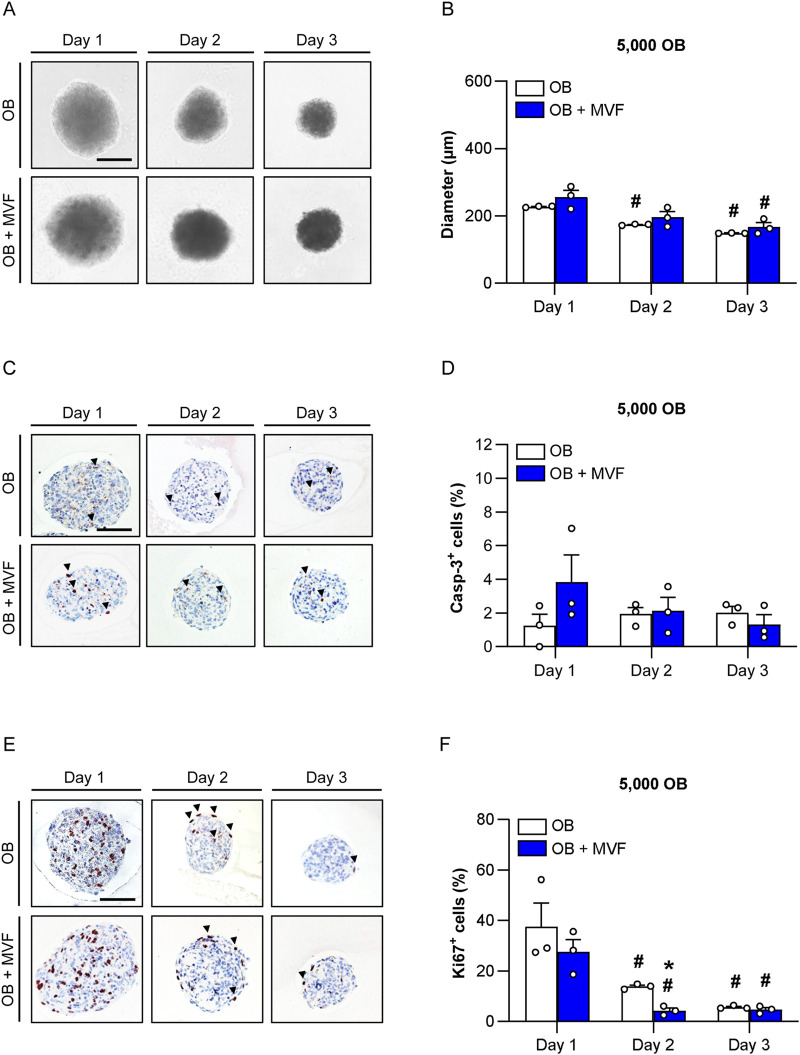
Size, viability and proliferative activity of spheroids consisting of 5,000 OB. **(A)** Microscopic images of OB and OB + MVF spheroids on day 1–3. Scale bar: 100 µm. **(B)** Diameter (µm) of OB and OB + MVF spheroids on day 1–3. Mean ± SEM (n = 3 each). ^#^ *p* < 0.05 vs. OB or OB + MVF spheroids on day 1. **(C,E)** Immunohistochemical detection of apoptotic casp-3^+^ cells (arrowheads) **(C)** and proliferating Ki67^+^ cells (arrowheads) **(E)** within OB and OB + MVF spheroids on day 1–3. Cell nuclei were stained with hematoxylin. Scale bars: C = 100 μm; E = 100 µm. **(D,F)** Casp-3^+^ cells (%) **(D)** and Ki67^+^ cells (%) **(F)** within OB and OB + MVF spheroids on day 1–3. Mean ± SEM (n = 3 each). ^*^ *p* < 0.05 vs. OB spheroids; ^#^ *p* < 0.05 vs. OB or OB + MVF spheroids on day 1.

Using the liquid overlay technique, it was possible to generate 3D mono-culture spheroids consisting of 5,000, 10,000 or 20,000 OB as well as co-culture spheroids containing a combination of the identical numbers of OB and 200 MVF under highly standardized conditions. During the generation process, the size of the spheroids was daily assessed by microscopic measurements of the spheroid diameters (Figures 1A, B; Supplementary Figure S1A; Supplementary Figure S2A). As expected, the diameters of OB + MVF spheroids were slightly larger on day 1 when compared to those of OB spheroids, however, without significant differences between the two groups (Figure 1B; Supplementary Figure S1B; Supplementary Figure S2B). Throughout the following observation period, the diameters of both OB and OB + MVF spheroids progressively decreased regardless of the number of incorporated OB. Accordingly, all spheroids finally exhibited a compact and round shape on day 3.

### 3.2 Viability of spheroids

Apoptotic cell death within the newly forming spheroids was analyzed by immunohistochemical detection of casp-3 (Figure 1C; Supplementary Figure S1C; Supplementary Figure S2C). OB and OB + MVF spheroids only contained 1%–4% of casp-3^+^ apoptotic cells over time without significant differences between the groups (Figure 1D; Supplementary Figure S1D; Supplementary Figure S2D). Of note, these apoptotic cells were randomly distributed within the spheroids without clustering in certain areas.

### 3.3 Proliferative activity of spheroids

Additional immunohistochemical stainings against Ki67 served for the analysis of cell proliferation within the spheroids (Figure 1E; Supplementary Figure S1E; Supplementary Figure S2E). On day 1, the spheroids of all groups exhibited a high proliferative activity with a fraction of ∼30–50% Ki67^+^ cells (Figure 1F; Supplementary Figure S1F; Supplementary Figure S2F). Throughout the following observation period, the fraction of Ki67^+^ cells significantly decreased to ∼5% on day 3 (Figure 1F; Supplementary Figure S1F; Supplementary Figure S2F). Of interest, this decline in cell proliferation was accelerated within co-culture spheroids, as indicated by significantly reduced numbers of Ki67^+^ cells in spheroids generated of 5,000 or 10,000 OB and MVF on day 2 when compared to OB spheroids (Figure 1F; Supplementary Figure S1F).

### 3.4 Microvascular network formation and surface topography of spheroids

**FIGURE 2:**
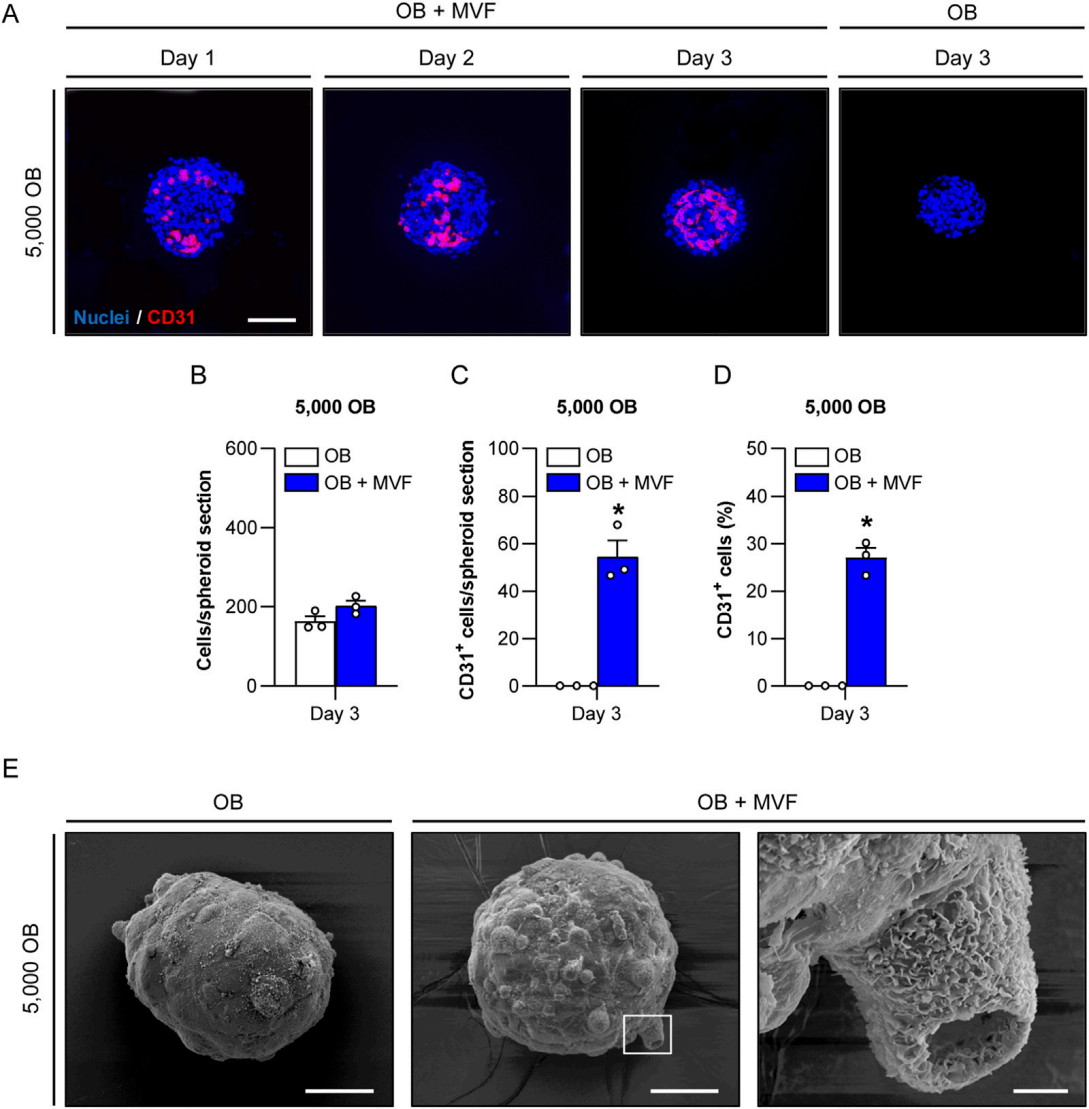
Microvascular network formation and surface topography of spheroids consisting of 5,000 OB. **(A)** Immunofluorescent detection of CD31^+^ endothelial cells in OB + MVF spheroids on day 1–3. OB spheroids without incorporated MVF served as controls. Cell nuclei were stained with Hoechst 33342. Scale bar: 100 µm. **(B–D)** Overall number of cells (per spheroid section) **(B)**, CD31^+^ cells (per spheroid section) **(C)** and CD31^+^ cells (%) **(D)** within OB and OB + MVF spheroids on day 3. Mean ± SEM (n = 3 each). ^*^ *p* < 0.05 vs. OB spheroids. **(E)** Surface topography of OB and OB + MVF spheroids on day 3, as visualized by scanning electron microscopy. The right panel shows a higher magnification of the white frame in the middle panel. Scale bars: Left and middle panel = 50 μm; right panel = 5 µm.

We next investigated microvascular network formation within the spheroids by immunohistochemical detection of the endothelial cell marker CD31 (Figure 2A; Supplementary Figure S3A, E). As expected, OB spheroids did not contain any endothelial cells. In contrast, the incorporated MVF in OB + MVF spheroids could easily be visualized by their strong expression of CD31. On day 1, they were randomly distributed between the CD31^−^ OB. In course of the spheroid maturation, the MVF reorganized and reassembled into new microvascular networks until day 3. However, this was only the case in small co-culture spheroids containing 5,000 OB, whereas the larger spheroids still presented with a random distribution of non-connected, individual MVF at this late time point (Figure 2A; Supplementary Figure S3A, E). Accordingly, co-culture spheroids containing 5,000 OB exhibited the highest number and fraction of incorporated CD31^+^ cells when compared to the larger spheroids (Figures 2B–D; Supplementary Figure S3B–H). Based on these findings, we only used spheroids generated of 5,000 OB with or without MVF for all further experiments.

To analyze the surface topography of OB and OB + MVF spheroids, we next performed scanning electron microscopy (Figure 2E). The surface of the spheroids was characterized by a heterogeneous surface pattern, which was more pronounced in the OB + MVF group. This was most probably due to the mixture of different cell types within these spheroids. Moreover, OB + MVF spheroids sporadically exhibited MVF with a clearly visible open lumen on their surface (Figure 2E).

### 3.5 Angiogenic activity of spheroids

**FIGURE 3:**
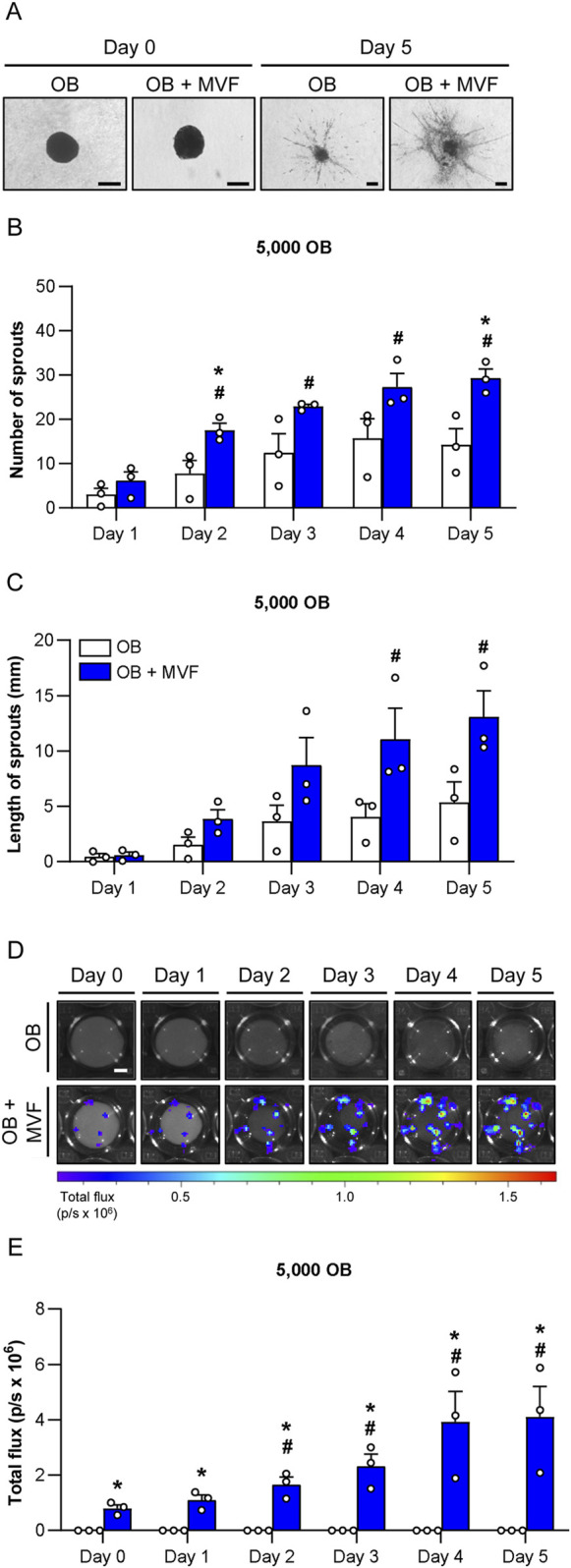
Angiogenic activity of spheroids consisting of 5,000 OB. **(A)** Microscopic images of sprouting OB and OB + MVF spheroids on day 0 and 5. Scale bar: 100 µm. **(B,C)** Number **(B)** and length **(C)** of sprouts of OB and OB + MVF spheroids on day 1–5. Mean ± SEM (n = 3 each). *p < 0.05 vs. OB spheroids; ^#^ *p* < 0.05 vs. OB or OB + MVF spheroids on day 1. **(D)** Bioluminescence images of sprouting OB and OB + MVF spheroids on day 0–5. Scale bar: 400 µm. **(E)** Total flux (p/s × 106) of bioluminescence imaging of OB and OB + MVF spheroids on day 0–5. Mean ± SEM (n = 3 each). *p < 0.05 vs. OB spheroids; ^#^ *p* < 0.05 vs. OB or OB + MVF spheroids on day 0.

To study the angiogenic activity of OB and OB + MVF spheroids, we performed a spheroid sprouting assay over 5 days (Figures 3A–E). In this assay, the spheroids were embedded in collagen gel and the length as well as the number of their outgrowing sprouts were daily assessed (Figures 3A–C). Bright field images showed that in both groups newly formed sprouts progressively grew out of the spheroids over time. However, the number and length of these sprouts were markedly increased in the group of OB + MVF spheroids when compared to OB spheroids (Figures 3A–C). Because the latter ones only existed of OB and did not contain any endothelial cells, it is obvious that their sprouts could only originate from individual OB migrating out of the spheroids into the surrounding collagen gel. Accordingly, these sprouts were also much thinner (Figure 3A). In contrast, OB + MVF spheroids exhibited real vessel sprouts, resulting from the angiogenic outgrowth of the incorporated MVF. This was proven by additional bioluminescence imaging (Figures 3D, E). In fact, in contrast to the group of OB spheroids, a progressively increasing total flux was measured in the group of co-culture spheroids containing MVF from luciferase^+^ FVB-Tg (CAG-luc-GFP)L2G85Chco/J donor mice (Figure 3E). Moreover, at later time points the bioluminescence signals were not only detected within the spheroids, but also in their surroundings, indicating vascular outgrowth into the collagen gel (Figure 3D). Taken together, these results show a high angiogenic activity of OB + MVF spheroids in contrast to OB spheroids.

### 3.6 Osteogenic differentiation of spheroids

**FIGURE 4:**
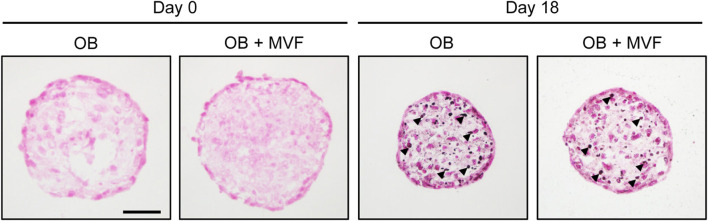
Osteogenic differentiation capacity of spheroids consisting of 5,000 OB. Microscopic images of von Kossa-stained OB and OB + MVF spheroids on day 0 and 18 after osteogenic differentiation. Arrowheads indicate mineralization. Scale bar: 50 µm.

To prove the osteogenic differentiation capacity of OB and OB + MVF spheroids, we cultivated spheroids containing 5,000 OB in medium supplemented with osteogenic differentiation factors for 18 days and subsequently assessed their mineralization by means of von Kossa staining. We detected first signs of mineralization in both OB and OB + MVF spheroids without marked differences between the two spheroid types (Figure 4). This indicates that the incorporation of MVF into the OB spheroids does not affect their osteogenic differentiation capacity.

### 3.7 Vascularization of transplanted spheroids

**FIGURE 5:**
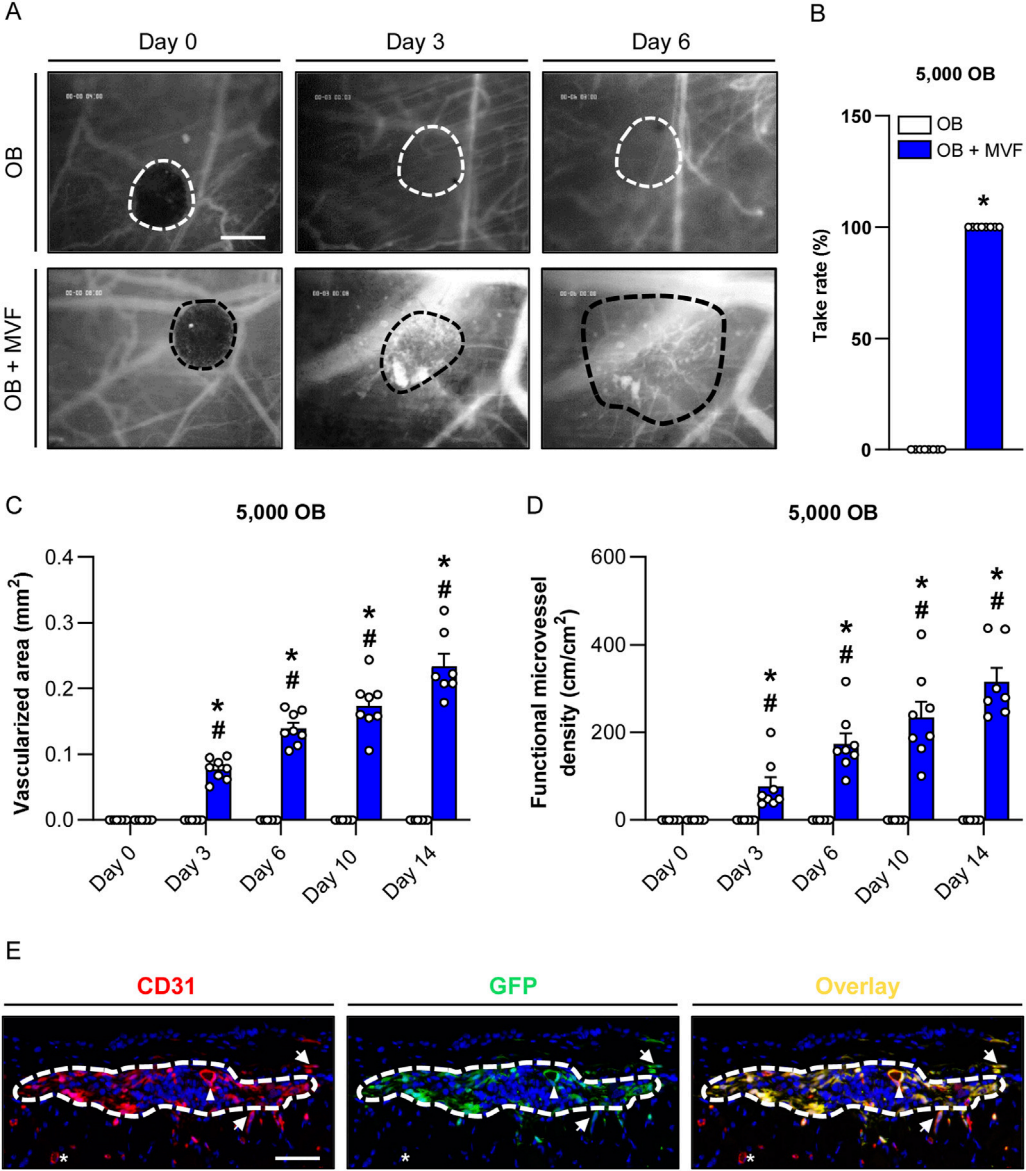
Vascularization of transplanted spheroids consisting of 5,000 OB. **(A)** Intravital fluorescent microscopic images of transplanted OB and OB + MVF spheroids within the dorsal skinfold chamber on day 0, 3 and 6. FITC-labeled dextran 150,000 was used for the visualization of blood-perfused microvessels. The borders of the grafts are marked by broken lines. Scale bar: 200 μm. **(B)** Take rate (%) of OB and OB + MVF spheroids on day 14 after transplantation into the dorsal skinfold chamber. Mean ± SEM (n = 8 each). ^*^ *p* < 0.05 vs. OB spheroids. **(C,D)** Vascularized area (mm^2^) **(C)** and functional microvessel density (cm/cm^2^) **(D)** of OB and OB + MVF spheroids on day 0, 3, 6, 10 and 14. Mean ± SEM (n = 8 each). *p < 0.05 vs. OB spheroids; ^#^ *p* < 0.05 vs. OB or OB + MVF spheroids on day 0. **(E)** Immunofluorescent detection of CD31^+^ endothelial cells and GFP^+^ cells within OB + MVF spheroids on day 14 after transplantation (arrowhead = CD31^+^/GFP^+^ microvessel within the graft; arrows = CD31^+^/GFP^+^ microvessels outside the graft; asterisk = CD31^+^/GFP^−^ microvessel outside the graft). Scale bar: 75 µm.

In a final set of *in vivo* experiments, we analyzed the vascularization capacity of OB and OB + MVF spheroids in the dorsal skinfold chamber model (Figures 5A–E). For this purpose, we transplanted 4 OB and 4 OB + MVF spheroids onto the striated skin muscle tissue within the chamber observation window. The quantitative analysis of microvascular network formation and microhemodynamic parameters within the grafts was performed after FITC-labeled dextran injection by means of intravital fluorescence microscopy. Of note, we could not detect any vascularization of transplanted OB spheroids over the entire observation period, which corresponds to a take rate of 0% (Figures 5A, B). In contrast, the incorporation of MVF into OB spheroids resulted in a take rate of 100% (Figure 5B). This was due to the fact that blood-perfused microvascular networks rapidly developed within OB + MVF spheroids between day 3 and 14 (Figure 5A). Accordingly, they exhibited a progressively larger vascularized area and higher functional microvessel density throughout the observation period (Figures 5C, D). The additional measurement of microhemodynamic parameters revealed that the centerline RBC velocity and volumetric blood flow of individual microvessels within the OB + MVF spheroids increased while their diameters slightly decreased over time (Table 1).

**TABLE 1:** Diameter (µm), centerline RBC velocity (µm/s) and volumetric blood flow (pL/s) of individual microvessels within OB and OB + MVF spheroids (containing 5,000 OB) on days 0, 3, 6, 10 and 14 after transplantation into the dorsal skinfold chamber.

	Day 0	Day 3	Day 6	Day 10	Day 14
Diameter (µm)					
OB	—	—	—	—	—
OB + MVF	—	11.6 ± 0.9	10.6 ± 1.2	10.3 ± 0.4	10.2 ± 0.5
Centerline RBC velocity (µm/s)					
OB	—	—	—	—	—
OB + MVF	—	40.7 ± 11.4	133.2 ± 19.8^#^	216.8 ± 18.3^#^	231.8 ± 17.0^#^
Volumetric blood flow (pL/s)					
OB	—	—	—	—	—
OB + MVF	—	3.4 ± 1.3	12.7 ± 3.2^#^	18.4 ± 2.4^#^	18.7 ± 2.4^#^

Mean ± SEM (n = 8 each). ^#^P < 0.05 vs. OB + MVF on day 3.

At the end of the *in vivo* experiments, the grafted OB + MVF spheroids were further analyzed by means of immunohistochemistry (Figure 5E). These analyses revealed that 100% of all microvessels within the grafts were CD31^+^/GFP^+^, proving their origin from the incorporated GFP^+^ MVF. Moreover, we also detected a few GFP^+^ microvessels in the surrounding host tissue, demonstrating that individual MVF even grew out of the spheroids further improving the vascularization at the transplantation site (Figure 5E).

## 4 Discussion

Scaffold-free bone tissue engineering based on spheroids as building blocks is an emerging approach to improve the treatment of critical-sized bone defects. In fact, spheroids better mimic the physiological milieu of natural bone in contrast to monolayer cultures due to their 3D structure and intensive cell-cell contacts (Elliott and Yuan, 2011). Moreover, spheroid-based bioassembly approaches offer the exciting opportunity to manufacture large-scale bone tissues (Prabhakaran et al., 2023). To provide a sufficient blood supply to such tissues, we herein introduce the biofabrication of prevascularized spheroids by fusion of OB with MVF. Our results demonstrate that this is an effective strategy, because the incorporated MVF rapidly reorganize into new functional microvascular networks within the spheroids and develop interconnections to the surrounding host microvasculature after transplantation.

Various methods for the biofabrication of spheroids have been established, including the culture of cells on low-attachment plates, magnetic levitation as well as the hanging drop or liquid overlay technique (Del Duca et al., 2004; Ivascu and Kubbies, 2006; Whatley et al., 2014; Zoetemelk et al., 2019). All of these methods exhibit distinct advantages and disadvantages. For instance, the use of low-attachment plates is expensive, whereas the hanging drop technique is cheaper but not suitable for the fabrication of larger spheroids, because the volume of the hanging drops is limited due to their surface tension (Wang and Yang, 2008). Furthermore, studies have demonstrated that magnetic levitation can disrupt F-actin stress fibers and microtubules of the cytoskeleton and induce apoptosis (Sytkowski and Davis, 2001; Meng et al., 2011). Moreover, it depends on the used cell line, which procedure offers the best conditions to form spheroids (Białkowska et al., 2020; Pinto et al., 2020). According to our good experiences in generating different spheroid types by means of the liquid overlay technique (Walser et al., 2013; Laschke et al., 2014; Nalbach et al., 2021a; Nalbach et al., 2021b), we herein also used this approach for the biofabrication of OB and OB + MVF spheroids. These spheroids were reproducible in size and shape and exhibited a high stability after 3 days, which enabled their easy handling during further *in vitro* and *in vivo* experiments.

To evaluate in a first step, which ratio between OB and MVF is most suitable for the biofabrication of prevascularized spheroids, we combined 5,000, 10,000 or 20,000 OB with 200 MVF each, whereas OB spheroids without MVF served as controls. Of note, all newly developing spheroids exhibited a comparably high cell viability. This is an unexpected result, because the size of spheroids crucially determines their survival, whereby larger spheroids are more prone to cell death due to insufficient oxygen diffusion into their core (Chen et al., 2018; Singh et al., 2020; Nalbach et al., 2021b).

Moreover, we found that the diameters of OB and OB + MVF spheroids decreased over time. This is a typical sign for the maturation of spheroids, which is associated with the formation of intense intercellular contacts (Metzger et al., 2011; Jennewein et al., 2016). It is well known that such contacts suppress cell proliferation, also referred to as contact inhibition (McClatchey and Yap, 2012; Ribatti, 2017). Accordingly, we detected a massive reduction of cell proliferation from 50% to 5% on day 1–3 within the spheroids of all groups. Of interest, this decline in cell proliferation was accelerated within co-culture spheroids consisting of 5,000 or 10,000 OB and MVF when compared to controls. Hence, it may be speculated that MVF promote the maturation of OB spheroids. However, this seems only to be the case for smaller spheroids, because this effect was not detected in co-culture spheroids consisting of 20,000 OB and MVF, most probably due to an unfavorable ratio between OB and MVF. In line with this view, we additionally found that MVF most effectively reorganized and reassembled into new microvascular networks in small co-culture spheroids containing 5,000 OB. Taken together, these findings indicate that the ratio of 5,000 OB and 200 MVF provides the best conditions for the generation of prevascularized spheroids for bone tissue engineering. Hence, we used spheroids with this composition for additional *in vitro* and *in vivo* experiments testing their functionality.

For this purpose, we first performed a well-established *in vitro* spheroid sprouting assay, which enables the analysis of sprout formation within a physiological 3D environment (Heiss et al., 2015). In the present study, we combined this assay for the first time with the technique of bioluminescence imaging by fusing MVF from luciferase^+^ FVB-Tg(CAG-luc-GFP)L2G85Chco/J donor mice with luciferase^−^ OB. This enabled us to prove the outgrowth of real angiogenic sprouts originating from luciferase^+^ MVF in the group of co-culture spheroids, whereas sprout formation in the group of OB spheroids could be traced back to the migration of individual luciferase^−^ OB into the surrounding collagen gel. In line with the fact that isolated MVF exhibit a high angiogenic activity (Nalbach et al., 2021a; Wrublewsky et al., 2022), our spheroid sprouting assay showed that the total number and length of sprouts were markedly higher in the group of OB + MVF spheroids when compared to OB spheroids. Notably, additional *in vitro* experiments proved that the incorporation of MVF does not affect the osteogenic differentiation capacity of OB + MVF spheroids. This indicates that these spheroids could be indeed useful for bone regeneration.

To further assess the vascularization of OB and OB + MVF spheroids *in vivo*, we used the mouse dorsal skinfold chamber model in combination with repeated intravital fluorescence microscopy. This approach is not only suitable to study the development of new microvessels in transplanted tissues, but also enables the assessment of their functionality and microhemodynamic characteristics by direct visualization of blood perfusion using the contrast agent FITC-labeled dextran (Nalbach et al., 2021a; Nalbach et al., 2021b). Of note, during the entire observation period, we could not detect any newly formed microvessels within grafted OB spheroids, which corresponds to a take rate of 0%. In contrast, OB + MVF spheroids rapidly vascularized over time and already exhibited completely blood-perfused areas on day 3 after transplantation. This is a typical sign of inosculation, i.e., the interconnection of MVF with the surrounding host microvasculature. Accordingly, our immunohistochemical analyses of the spheroids on day 14 showed that 100% of all microvessels within the grafts were CD31^+^/GFP^+^, proving their origin from the incorporated GFP^+^ MVF. Moreover, we found that the diameters of the microvessels inside the spheroids decreased while the centerline RBC velocity and volumetric blood flow increased over time, which indicates the progressive organization, stabilization and remodeling of the new microvascular networks within the grafts (Später et al., 2018).

Taken together, we herein introduced a novel approach for the biofabrication of prevascularized spheroids for bone tissue engineering. In fact, we found that the fusion of MVF and OB results in viable spheroids with a high vascularization capacity, which rapidly inosculate after transplantation. Because a sufficient vascularization is a major prerequisite for adequate bone formation (Simunovic and Finkenzeller, 2021), these spheroids represent promising building blocks for larger bone constructs. However, it should be considered that adequate bone regeneration seems to be crucially dependent on a well-balanced temporal and spatial vascularization of bone defects (Menger et al., 2022). In fact, several preclinical and clinical studies indicate that non-unions are sometimes also considerably well vascularized although they do not heal (Reed et al., 2002; Garcia et al., 2008). Moreover, it has been shown that too much vascularization may not promote bone defect healing but may even result in healing failure (Orth et al., 2018; Ruehle et al., 2019). Based on the results of the present proof-of-concept study, we cannot exclude such a potential, counter-productive effect of OB + MVF spheroids due to their high vascularization capacity. Therefore, OB + MVF spheroids should be next tested in appropriate *in vivo* bone defect models.

## Data Availability

The raw data supporting the conclusions of this article will be made available by the authors, without undue reservation.
