# Supplementary material for: Biofabrication of prevascularized spheroids for bone tissue engineering by fusion of microvascular fragments with osteoblasts
Source: Front Bioeng Biotechnol. 2024 Sep 10;12:1436519. doi: 10.3389/fbioe.2024.1436519 (PMC11419975; doi:10.3389/fbioe.2024.1436519)
Supplement: Supplementary file 1 [file DataSheet1.PDF]

Supplementary Material

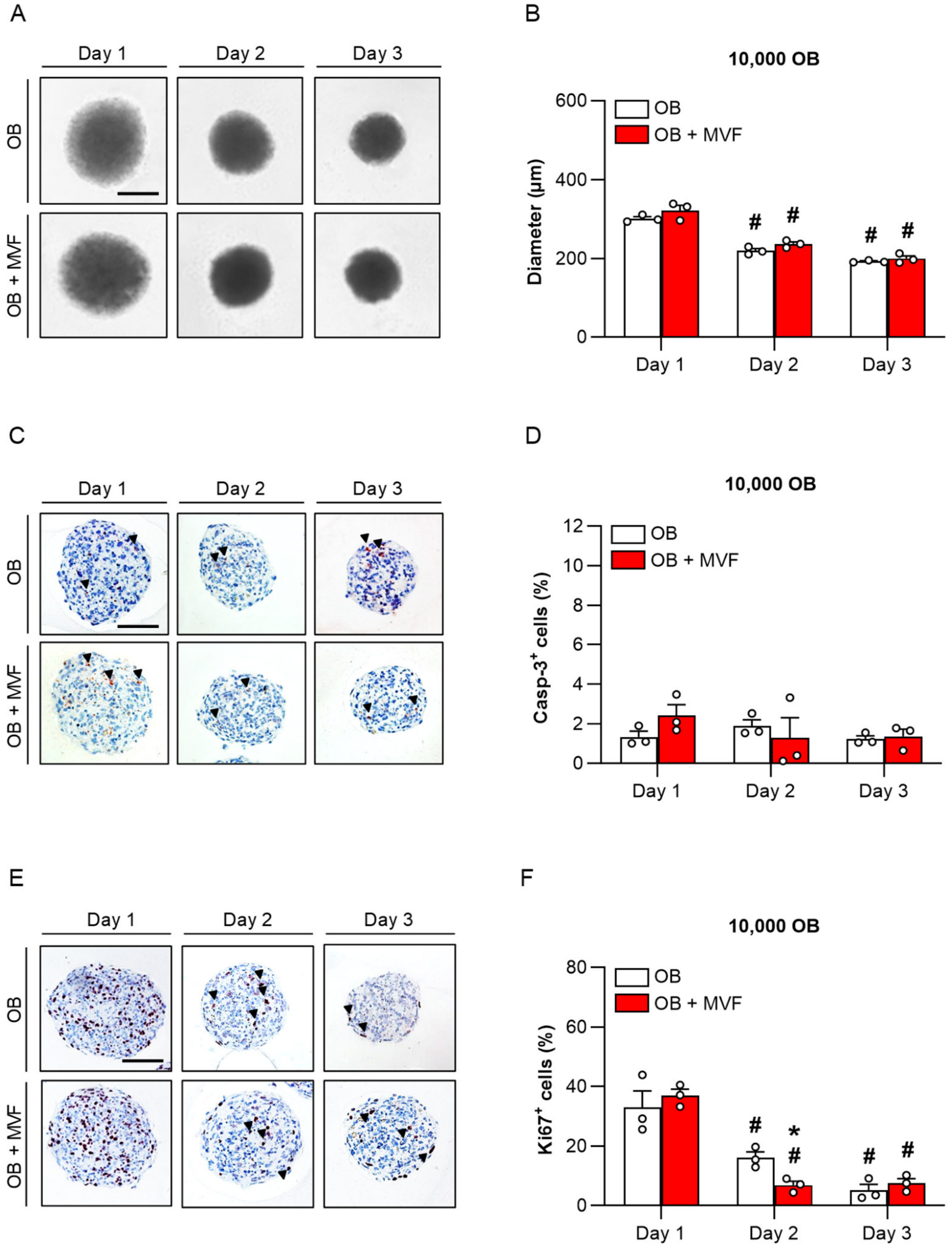

Supplementary Figure S1. Size, viability and proliferative activity of spheroids consisting of 10,000 OB. (A) Microscopic images of OB and OB + MVF spheroids on day 1-3. Scale bar: 175 µm. (B) Diameter (µm) of OB and OB + MVF spheroids on day 1-3. Mean ± SEM (n = 3 each). $^{\#}P < 0.05$ vs.

OB or OB + MVF spheroids on day 1. (C, E) Immunohistochemical detection of apoptotic casp-3$^+$ cells (arrowheads) (C) and proliferating Ki67$^+$ cells (arrowheads) (E) within OB and OB + MVF spheroids on day 1-3. Cell nuclei were stained with hematoxylin. Scale bars: C = 175 µm; E = 150 µm. (D, F) Casp-3$^+$ cells (%) (D) and Ki67$^+$ cells (%) (F) within OB and OB + MVF spheroids on day 1-3. Mean ± SEM (n = 3 each). $^*P < 0.05$ vs. OB spheroids; $^\#P < 0.05$ vs. OB or OB + MVF spheroids on day 1.

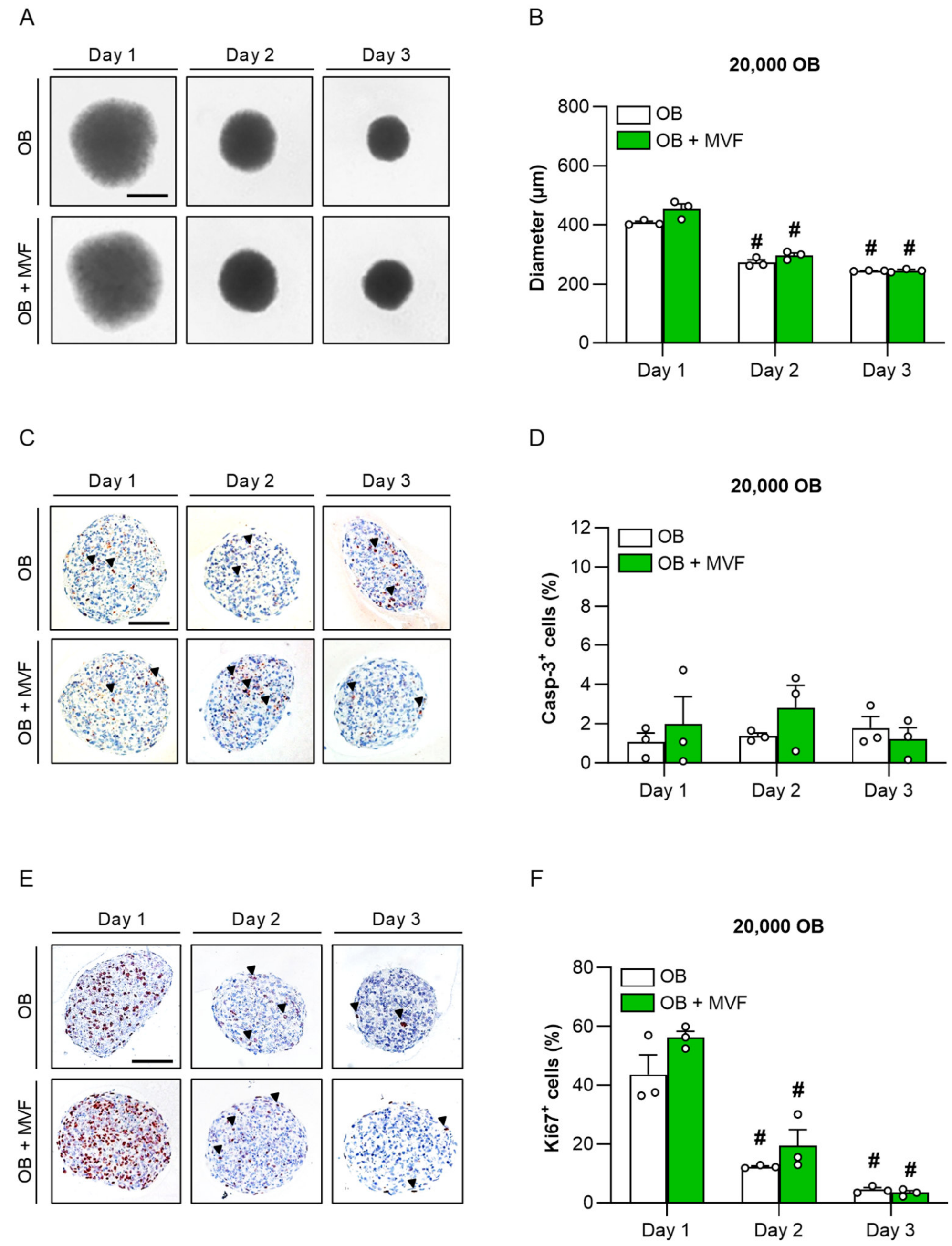

Supplementary Figure S2. Size, viability and proliferative activity of spheroids consisting of 20,000 OB. (A) Microscopic images of OB and OB + MVF spheroids on day 1-3. Scale bar: 200 µm. (B) Diameter (µm) of OB and OB + MVF spheroids on day 1-3. Mean ± SEM (n = 3 each). $^{\#}P < 0.05$ vs. OB or OB + MVF spheroids on day 1. (C, E) Immunohistochemical detection of apoptotic casp-3^{+} cells (arrowheads) (C) and proliferating Ki67^{+} cells (arrowheads) (E) within OB and OB + MVF spheroids on day 1-3. Cell nuclei were stained with hematoxylin. Scale bars: C = 200 µm; E = 200 µm. (D, F) Casp-3^{+} cells (%) (D) and Ki67^{+} cells (%) (F) within OB and OB + MVF spheroids on day 1-3. Mean ± SEM (n = 3 each). $^{\#}P < 0.05$ vs. OB or OB + MVF spheroids on day 1.

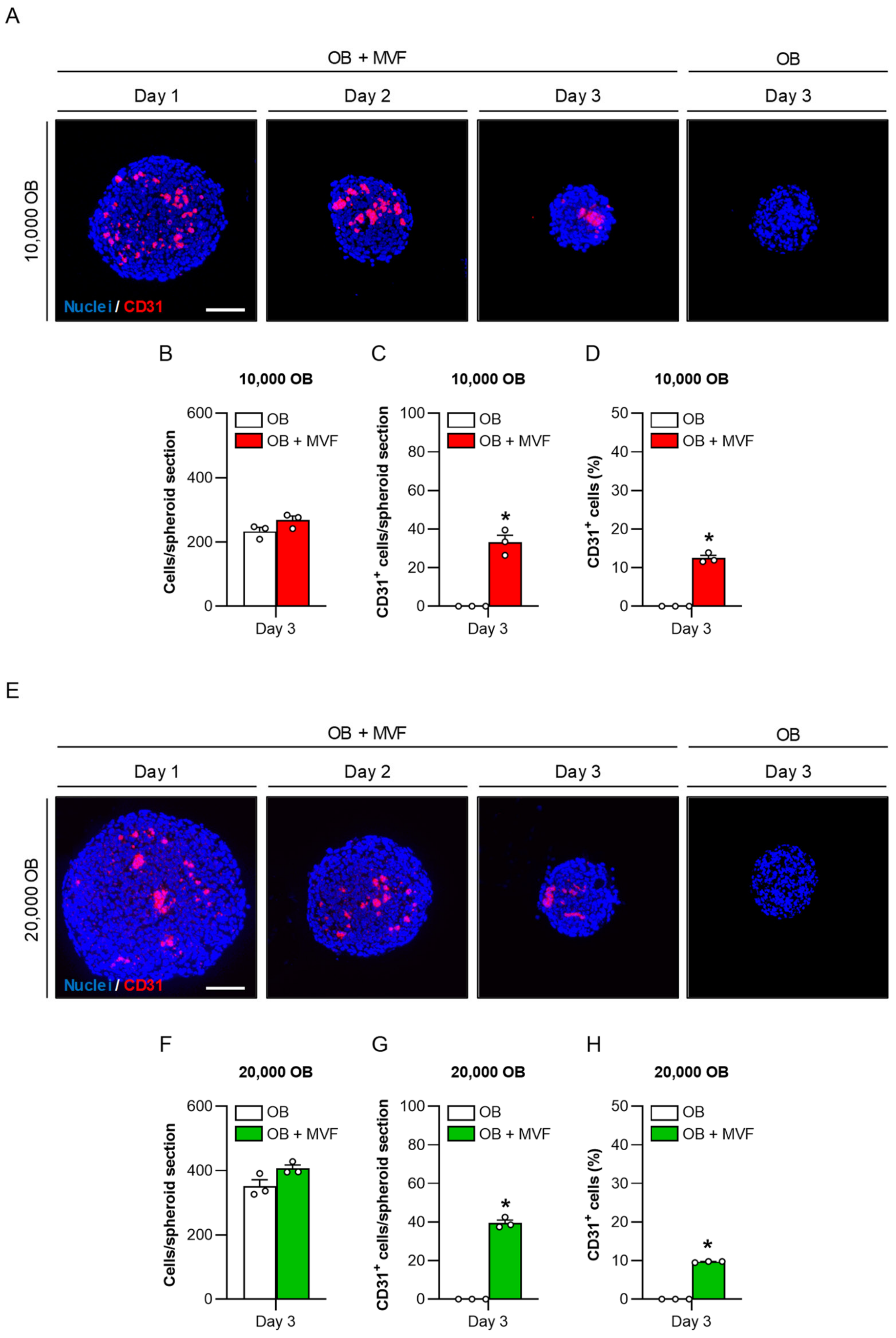

Supplementary Figure S3. Microvascular network formation and surface topography of spheroids consisting of 10,000 and 20,000 OB. (A) Immunofluorescent detection of $CD31^{+}$ endothelial cells in OB + MVF spheroids consisting of 10,000 OB on day 1-3. OB spheroids without incorporated MVF served as controls. Cell nuclei were stained with Hoechst 33342. Scale bar: 100 µm. (B-D) Overall

number of cells (per spheroid section) (B), $CD31^+$ cells (per spheroid section) (C) and $CD31^+$ cells (%) (D) within OB and OB + MVF spheroids on day 3. Mean ± SEM (n = 3 each). $^*P < 0.05$ vs. OB spheroids. (E) Immunofluorescent detection of $CD31^+$ endothelial cells in OB + MVF spheroids consisting of 20,000 OB on day 1-3. OB spheroids without incorporated MVF served as controls. Cell nuclei were stained with Hoechst 33342. Scale bar: 100 µm. (F-H) Overall number of cells (per spheroid section) (F), $CD31^+$ cells (per spheroid section) (G) and $CD31^+$ cells (%) (H) within OB and OB + MVF spheroids on day 3. Mean ± SEM (n = 3 each). $^*P < 0.05$ vs. OB spheroids.
